# NEO212, a Perillyl Alcohol-Temozolomide Conjugate, Triggers Macrophage Differentiation of Acute Myeloid Leukemia Cells and Blocks Their Tumorigenicity

**DOI:** 10.3390/cancers14246065

**Published:** 2022-12-09

**Authors:** Thomas C. Chen, Radu O. Minea, Steve Swenson, Zhuoyue Yang, Thu Zan Thein, Axel H. Schönthal

**Affiliations:** 1 Department of Neurosurgery, Keck School of Medicine, University of Southern California (USC), Los Angeles, CA 90089, USA; 2 USC Norris Comprehensive Cancer Center, Los Angeles, CA 90033, USA; 3 Department of Molecular Microbiology & Immunology, Keck School of Medicine, University of Southern California, Los Angeles, CA 90089, USA

**Keywords:** acute myeloid leukemia, cytarabine resistance, DNA alkylation, perillyl alcohol, temozolomide

## Abstract

**Simple Summary:**

Acute myeloid leukemia (AML) is a cancer of the blood and bone marrow, where cancer cells are unable to complete the maturation process towards white blood cells, but instead continue to proliferate. We are developing a novel chemotherapeutic drug, NEO212, that has shown anticancer activity in different preclinical cancer models, including AML. In the current study, we demonstrate that NEO212 forces AML cells to resume the differentiation process and progress towards the macrophage phenotype, which is accompanied by a loss of proliferation. NEO212 is able to achieve this growth-inhibitory effect even in AML cells that are resistant to other chemotherapeutic drugs in clinical use, such as cytarabine and temozolomide. In mouse models with AML, we found that treatment with NEO212 was well tolerated and resulted in an apparent cure of these animals. We propose that NEO212 should be developed further and evaluated in clinical trials with AML patients.

**Abstract:**

Many patients with acute myeloid leukemia (AML) are still dying from this disease. In the past, the alkylating agent temozolomide (TMZ) has been investigated for AML and found to be partially effective; however, the presence of O6-methylguanine DNA methyltransferase (MGMT; a DNA repair enzyme) in tumor cells confers profound treatment resistance against TMZ. We are developing a novel anticancer compound, called NEO212, where TMZ was covalently conjugated to perillyl alcohol (a naturally occurring monoterpene). NEO212 has revealed robust therapeutic activity in a variety of preclinical cancer models, including AML. In the current study, we investigated its impact on a panel of human AML cell lines and found that it exerted cytotoxic potency even against MGMT-positive cells that were highly resistant to TMZ. Furthermore, NEO212 strongly stimulated the expression of a large number of macrophage-associated marker genes, including CD11b/ITGAM. This latter effect could not be mimicked when cells were treated with TMZ or an equimolar mix of individual agents, TMZ plus perillyl alcohol. The superior cytotoxic impact of NEO212 appeared to involve down-regulation of MGMT protein levels. In a mouse model implanted with TMZ-resistant, MGMT-positive AML cells, two 5-day cycles of 25 mg/kg NEO212 achieved an apparent cure, as mice survived >300 days without any signs of disease. In parallel toxicity studies with rats, a 5-day cycle of 200 mg/kg NEO212 was well tolerated by these animals, whereas animals that were given 200 mg/kg TMZ all died due to severe leukopenia. Together, our results show that NEO212 exerts pleiotropic effects on AML cells that include differentiation, proliferation arrest, and eventual cell death. In vivo, NEO212 was well tolerated even at dosages that far exceed the therapeutic need, indicating a large therapeutic window. These results present NEO212 as an agent that should be considered for development as a therapeutic agent for AML.

## 1. Introduction

Despite progress in the treatment of acute myeloid leukemia (AML), the clinical outcome remains suboptimal and many patients are still dying from this disease. AML progresses rapidly and becomes fatal within months if not treated. First-line treatment consists of chemotherapy, which typically is separated into an induction and consolidation (postremission) phase. The induction phase generally uses cytarabine (AraC) in combination with an anthracycline such as daunorubicin, or high-dose AraC alone, although more recently alternative treatments have been introduced, primarily for “fit” patients with better performance status [1,2]. After complete remission is achieved, drug composition of the subsequent consolidation therapy is variable and individualized based on prognostic factors, including tumor cytogenetics and general health of the patient, and may include further chemotherapy and stem cell transplantation. In addition, novel targeted therapies can be considered, such as the BH3-mimetic venetoclax, inhibitors of fms-like tyrosine kinase 3 (FLT3) or of isocitrate dehydrogenase 1 and 2 (IDH1, 2), and others. However, despite these complex therapeutic regimens, relapsed and refractory leukemia remains a challenge and better treatments are needed [3,4,5].

We are developing a novel cancer therapeutic molecule, called NEO212, which has been generated by covalent conjugation of two known anticancer agents, temozolomide (TMZ) and perillyl alcohol (POH) [6,7,8]. TMZ is an alkylating agent that represents the standard of chemotherapeutic care, along with radiation, for patients with glioblastoma (GBM) [9]. It alkylates several base moieties in DNA, but it is primarily the methylation of O6-guanine that mediates its decisive cytotoxic impact. This narrowly defined target makes the therapeutic benefit of TMZ reliant on the absence of 6-methylguanine DNA methyltransferase (MGMT), a DNA repair enzyme that efficiently removes methyl groups from the O6-position of guanine [10]. In essence, MGMT neutralizes the key cytotoxic impact of TMZ treatment. For example, the landmark EORTC/NCIC phase III trial by Stupp et al. [11], which investigated the addition of TMZ to radiation therapy (RT) in newly diagnosed GBM patients, established a significant survival benefit of TMZ, but only in patients with MGMT-negative tumors. In patients whose tumors lacked MGMT expression, the addition of TMZ to RT extended the median overall survival (OS) by 6.4 months as compared to RT alone, whereas in patients with MGMT-positive tumors this extension was less than 1 month [12]. As a consequence, the application of TMZ in GBM patients with MGMT-positive tumor tissue is considered unproductive and generally not recommended [10,13].

Beyond MGMT expression, several additional mechanisms of TMZ resistance have been recognized. Not surprisingly, other DNA repair systems play a key role as well, and these include mismatch repair (MMR) and base excision repair as key contributors, based on their activities directed at repairing the various methylated nucleobases that exist after TMZ treatment [10,14]. More recent studies have introduced autophagy, aberrant signaling pathways, epigenetic modifications, extracellular vesicle production, microRNAs, and microenvironmental influences as newly recognized mechanisms that may contribute to TMZ resistance [15,16].

Besides its established use for GBM, TMZ has also been evaluated for the treatment of hematological diseases. A few case studies reported on patients that responded well to TMZ treatment (e.g., [17,18]). Several clinical trials with AML patients, where TMZ was given as monotherapy or in combination with cisplatin, laromustine, or PARP inhibitors, also indicated therapeutic activity [19,20,21,22,23,24,25]. Several patients presented with complete responses, although it was also recognized that the best response to TMZ is dependent on low, or absent, expression of MGMT in patients’ tumor cells [20,21,22,25]. The protective effect of MGMT against TMZ is particularly detrimental in AML, because the majority of AML patients present with significant MGMT expression in their tumor tissue [26], consistent with laboratory studies that only 25% of leukemia cells harbor low levels of MGMT [27], and inhibition of MGMT in vitro greatly sensitizes leukemia cells to TMZ [28].

POH is a naturally occurring monoterpene related to limonene that is found in the essential oils of a variety of botanicals, such as citrus fruit peel, lavender flowers, spearmint leaves, cranberries, and celery seeds [29]. While many preclinical studies established and characterized its anticancer potency, clinical trials with an oral formulation of POH in patients with solid cancer types were unable to establish clear therapeutic activity, and dose-limiting gastrointestinal side effects were observed [30,31]. More recently, however, a novel intranasal formulation of POH, also called NEO100, was studied in recurrent GBM patients; it was much better tolerated and showed encouraging activity [32,33,34].

Our rationale for covalently conjugating POH to TMZ, yielding NEO212, was based on in silico studies that predicted improved penetration of tumor tissues and biological barriers of the new agent [31,35]. Subsequent studies in different preclinical tumor models, including brain-metastatic breast cancer, glioblastoma, melanoma and others, demonstrated promising therapeutic activity of NEO212 [6,7,8,36,37]. We also investigated the effects of NEO212 in mouse models of AML, where we established proof of principle of striking therapeutic activity of NEO212 in AraC-resistant AML [38]. In the current study, we have zeroed in on the molecular and cellular mechanisms by which NEO212 achieves its potentially curative impact on drug-resistant AML in vitro and in vivo, with particular emphasis on the role of MGMT and the involvement of the macrophage differentiation process.

## 2. Materials and Methods

### 2.1. Pharmacologic Agents

NEO212 is a crystalline powder. It was manufactured by Norac Pharma (Azusa, CA, USA) under current good manufacturing practice (cGMP) conditions, and by Axon MedChem (Groningen, The Netherlands). Both products were kindly provided by NeOnc Technologies (Los Angeles, CA, USA) and resulted in identical experimental results. NEO212 was dissolved in DMSO (Santa Cruz Biotechnology, Dallas, TX, USA) at 100 or 500 mM for in vitro or in vivo experiments, respectively. Temozolomide was purchased from Sigma-Aldrich (St. Louis, MO, USA) and dissolved in DMSO to a concentration of 100 mM. Stock solutions of NEO212 and TMZ were stored at −80 °C. Perillyl alcohol was provided by NeOnc Technologies as NEO100 (Norac Pharma), which is a highly pure, cGMP-manufactured, pharmaceutical-grade version of POH that is in clinical use [33]. It is an oily liquid that was stored in a brown bottle at 4 °C. Immediately before use, it was diluted 1:12 with DMSO to produce a 600 mM stock solution. Further dilutions were carried out with cell culture medium as the diluent. O6-benzylguanine (O6BG; Santa Cruz Biotechnology) was dissolved in DMSO to 50 mM. 12-O-tetradecanoyl-phorbol-13-acetate (TPA; Sigma-Aldrich) was dissolved in DMSO to 20 mM. In all cases of cell treatment, the final DMSO concentration in the culture medium never exceeded 0.2%, and in fact was much lower in most cases.

### 2.2. Cell Culture

Human leukemia cell lines U937, THP1, KG1 and HL60 were obtained from the American Tissue Culture Collection (ATCC; Manassas, VA, USA). A cytarabine-resistant U937-derived cell line, 6D10, was generously provided by David Largaespada’s lab (U. Minnesota, Minneapolis, MN, USA) [39]. All cells were propagated in RPMI medium supplemented with 10% fetal bovine serum (FBS). Growth medium was supplemented with 100 U/mL penicillin and 0.1 mg/mL streptomycin. Penicillin, streptomycin, and RPMI (prepared with raw materials from Cellgro/MediaTech, Manassas, VA, USA) were provided by the Cell Culture Core lab of the USC/Norris Comprehensive Cancer Center. Cells were kept in a humidified incubator at 37 °C and a 5% CO_2_ atmosphere. FBS was obtained from Omega Scientific (Tarzana, CA, USA) and from X&Y Cell Culture (Kansas City, MO, USA). U937, THP1, KG1 and HL60 cells were passaged for less than 6 months after receipt, thus representing authenticated cells.

### 2.3. MTT Assay

Methylthiazoletetrazolium (MTT) assays were performed as detailed elsewhere [37]. Briefly, cells were seeded into 96-well plates at different densities ranging from 1–10 × 10^4^ per mL. Various concentrations of drug (or vehicle) were added and the cells were incubated for different lengths of time (48–120 h). Relatively long incubation times were necessary because the cell death mechanisms triggered by TMZ and NEO212 require two rounds of cell division to take effect [38,40]. At the end of the incubation period, MTT (Sigma-Aldrich) was added for 4 h, followed by solubilization solution and measurement of optical density. In individual experiments, each treatment condition was set up in duplicate or triplicate, and each experiment was repeated several times independently. All treatment conditions were applied to different cell densities, and toxic drug effects in general were somewhat stronger in cell cultures at lower density, although this had no impact on the qualitative differences between the different drugs used.

### 2.4. Immunoblots

Total cell lysates were prepared and analyzed by Western blot as described previously [37]. We used the following antibodies. Rabbit monoclonal MGMT antibody (#2739) from Cell Signaling Technology (Danvers, MA, USA) and mouse monoclonal actin antibody (SC-8432) from Santa Cruz Biotechnology. As secondary antibodies, we used horseradish peroxidase-conjugated, affinity-purified anti-rabbit (#111-035-003) or goat anti-mouse (#115-035-003) polyclonal IgG from Jackson ImmunoResearch (West Grove, PA, USA). All antibodies were used according to suppliers’ recommendations. For detection, SuperSignal West Pico PLUS Chemiluminescent Substrate was used (Thermo Fisher Scientific, Waltham, MA, USA). Immunoblots were repeated to confirm the results. 

### 2.5. Fluorescence-Activated Cell Sorting

Cells were seeded in 10 cm dishes at 2 × 10^5^ cells/mL, followed by drug treatment. At the end of the drug incubation period, cell cultures were transferred to 15-mL conical tubes and gently centrifuged for 5 min. The cell pellets were resuspended in 1 mL of fresh complete medium and incubated for 40 min at 37 °C with either an APC-labeled human CD11b (clone ICRF44) antibody or an APC-labeled IgG1, κ (clone MOPC-21) isotype control (BD Biosciences, Franklin Lakes, NJ, USA) per manufacturer’s recommendations. At the end of the incubation time, the cells were pelleted again and resuspended in ice-cold PBS supplemented with 10% FBS. FACS analysis was performed on an Aria I flow cytometer (BD Biosciences) equipped with four laser lines, including a red (633 nm) laser.

### 2.6. Reverse Transcription Quantitative Polymerase Chain Reaction (RT-qPCR)

Following treatment with drug, vehicle, or no treatment in vitro, cells were harvested and total RNA was isolated using Quick-RNA Miniprep Kit (Zymo Research, Irvine, CA, USA) according to the manufacturer’s protocol. RNA concentration was determined with NanoDrop 2000/2000c Spectrophotometer (Thermo Scientific). Quantitative RT-PCR was performed with iTaq Universal SYBR Green One-Step Kit (Bio-Rad Laboratories, Hercules, CA, USA) according to the manufacturer’s protocol. To amplify CD11b/ITGAM, we used the following primers: forward, 5′-CAGCATCAATATCAGGTCAGCA-3′; reverse, 5′-GAAGCTCAGCCAGAAAGTCG-3′. As internal control, we amplified glyceraldehyde-3-phosphate dehydrogenase (GAPDH) with primers: forward, 5′-TGACTTCAACAGCGACACCCA-3′; reverse, 5′-CACCCTGTTGCTGTAGCCAAA-3′. All primers were obtained from Integrated DNA Technologies (IDT, Coralville, IA, USA) as RxnReady primer pools, were 2 oligos are premixed in a single tube. These primers were resuspended in DNase/RNase-free water to a 100 µM stock solution, followed by 1:20 dilution to obtain the working concentration 5 µM. The reaction mix was 6.13 µL, composed of 0.13 µL reverse transcriptase, 5 µL SYBR Green, and 1 µL of 5 µM primers. Purified RNA was diluted with DNase/RNase-free water to 200 ng/3.9 µL, and 3.9 µL were added to the reaction mix. The resulting total was 10 µL for each reaction, which was added to a 96-well PCR plate. RT-PCR conditions were 50 °C for 2 min, then 95 °C for 10 min, followed by 40 cycles at 95 °C for 15 s and 60 °C for 1 min. The products were analyzed on an Applied Biosystems 7500 Fast Real-Time PCR System (Thermo Fisher) using Standard 7500 Mode. The relative expression levels of different samples were calculated by the 2^–ΔΔCt^ (Livak) method [41].

### 2.7. RNA Sequencing (RNA-Seq)

Cells were harvested by centrifugation and washing in phosphate-buffered saline (PBS), followed by transfer to 1.5-mL microcentrifuge tubes. “Dry” cell pellets were flash-frozen and shipped on dry ice to Novogene (Sacramento, CA, USA) for commercial processing of the samples, including quality controls, library construction, and RNA sequencing on Illumina sequencing platforms (Illumina, San Diego, CA, USA). The results were subjected to principal component analysis, heat map display, and differential gene expression analysis.

### 2.8. In Vivo Experiments

All animal experiments were reviewed and approved by the Institutional Animal Care and Use Committee (IACUC) of the University of Southern California (USC). For the implantation of human tumor cells into mice, we purchased immune-deficient, female 6–8-week-old NOD-SCID mice from the Jackson Laboratory (Bar Harbor, ME, USA). To determine toxic side effects of high-dose NEO212 and temozolomide, we used 29-week-old, female Fisher 344 rats, weighing about 200 g, that were obtained from Charles River Laboratories (Wilmington, MA, USA). All animals were housed at the USC Medical Center Animal Facility, which is AAALAC and AALAS certified and has written animal welfare assurance with the NIH-OLAW (Office of Laboratory Animal Welfare) that commits the institution to follow the standards established by the Animal Welfare Act.

#### 2.8.1. Experiments in Mice

For AML cell implantation into mice, we injected 5 × 10^4^ tumor cells in a volume of 50 µL 0.9% NaCl into the peritoneum, as described by others previously [42,43]. Several days later, mice received treatment via oral gavage with 25 mg/kg NEO212 or vehicle only. Treatment was for two or three cycles, where one cycle consisted of 5 consecutive days of once-daily dosing, followed by several days of a treatment holiday. Animals were observed and cared for on a daily basis.

#### 2.8.2. Experiments in Rats

Rats received 100 or 200 mg/kg NEO212 or TMZ for one cycle, where one cycle consisted of 5 consecutive days of once-daily dosing. Animals were observed daily and blood was collected. Further analysis (complete blood count with differential) was performed by Antech Diagnostics (Fountain Valley, CA, USA).

### 2.9. Statistical Analysis

All parametric data were analyzed using Prism 9 software (GraphPad Software, San Diego, CA, USA). Student *t*-tests were applied to calculate the significance values. Kaplan–Meier survival probability analysis was done with log-rank (Mantel-Cox) test. A probability value (*p*) < 0.05 was considered statistically significant.

## 3. Results

### 3.1. NEO212 Is Cytotoxic against AML Cells In Vitro

**Figure 1:**
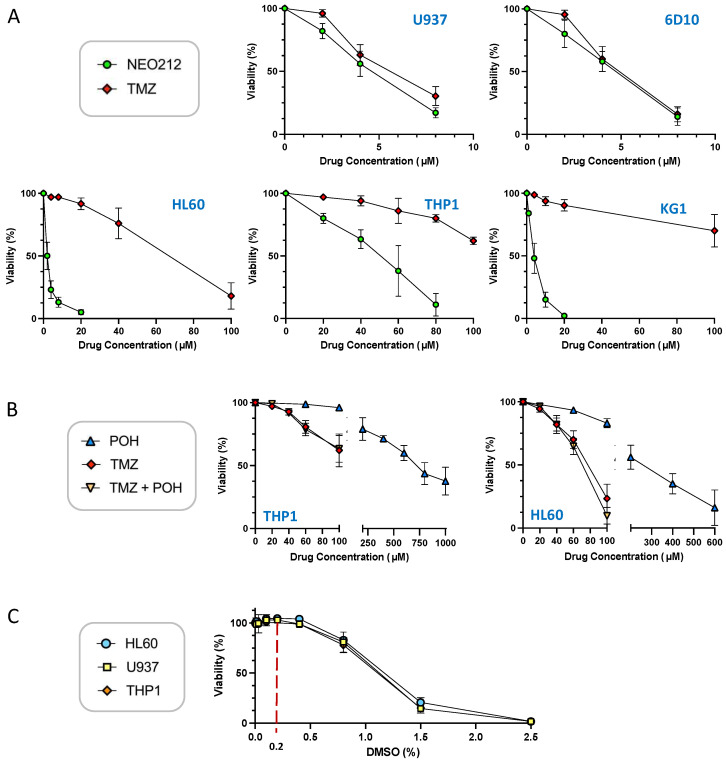
NEO212 is cytotoxic to AML cell lines. U937, 6D10, HL60, KG1, and THP1 cells were exposed to different drug treatment conditions, and cell viability was determined 5 days later by MTT assay. (**A**) Cells were treated with increasing concentrations of NEO212 or TMZ. (**B**) Cells were treated with POH alone, TMZ alone, or POH combined with TMZ. (**C**) Cells were exposed to increasing concentrations of DMSO. All values were normalized to vehicle control at the same time point.

We characterized the anticancer effects of NEO212 with the use of established AML cell lines U937, THP1, KG1, HL60, and 6D10, a well-characterized [39] highly AraC-resistant subline of U937. To start with, we performed standard in vitro MTT cytotoxicity assays, where cell viability in response to NEO212 treatment was compared to that after TMZ treatment. As shown in Figure 1A, U937, KG1, HL60, and 6D10 cells were effectively killed by NEO212, with an IC50 of about 5 µM or below. THP1 cells required noticeably higher concentrations, with an IC50 around 50 µM. Treatment with TMZ was similarly effective in U937 and 6D10 cells, but was much less potent in HL60, KG1, and THP1 cells. In HL60 cells, the IC50 of TMZ was >10-fold higher than the IC50 of NEO212, and in KG1 and THP1 cells TMZ did not reach IC50 at 100 µM, which was the highest concentration we used in these assays.

Based on the conjugated nature of NEO212, i.e., its composition of two units (TMZ and POH) where each one individually has been shown to exert anticancer activity on its own, we investigated whether a simple mix of these two molecules, as individual agents, would be able to mimic the potency of the conjugated NEO212 product. As presented in Figure 1B, this was not the case. When TMZ and POH were added to cells at equimolar concentrations, the cytotoxic IC50 was not decreased as compared to that of TMZ alone. For example, combination treatment of cells with 50 µM TMZ mixed with 50 µM POH was unable to lower the IC50 of 50 µM TMZ alone, i.e., there was no enhancing effect. The IC50 of POH alone was in the range of 300–600 µM, which is consistent with past studies showing that relatively high concentrations are generally required for this compound to deliver its cytotoxic impact. In all, these results demonstrate that NEO212 was strikingly more potent than the sum of its parts.

To perform cell treatments, the drugs we used were dissolved in DMSO, which sometimes is considered an inert vehicle. However, the literature contains many examples of biological effects of this chemical, including in cultured AML cell lines. It was therefore relevant to determine whether DMSO alone exerted any effects in our system. U937, HL60, and THP1 cells were treated with increasing concentrations of DMSO and cell viability was determined by MTT assay as above. As shown in Figure 1C, DMSO reduced cellular viability starting at around 0.8% concentration, and at 1.5% it killed most of the cells. However, at concentrations up to 0.4% it did not show a measurable negative impact on cell viability. Because the DMSO concentration range used in our experiments was from 0.02 to 0.2%, we deemed the potential impact of this vehicle as negligible.

### 3.2. NEO212 Overcomes MGMT-Mediated Drug Resistance

**Figure 2:**
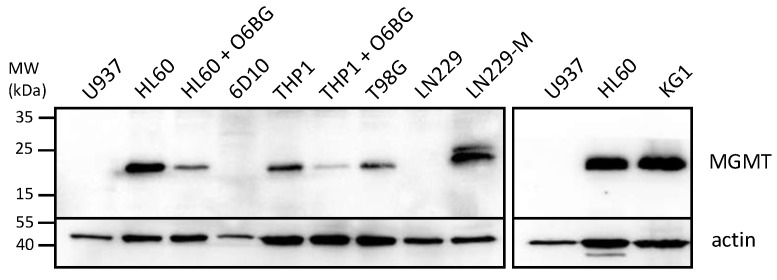
AML cells express different levels of MGMT. Cells grown in log phase were harvested and cell lysates were analyzed by Western blot analysis with an antibody specific for MGMT. Actin was used as the loading control. The following controls were included to ascertain specificity of the MGMT antibody: (i) HL60 and THP1 cells were treated for 18 h with 15 µM O6BG, an MGMT inhibitor known to down-regulate MGMT protein levels; (ii) T98G are glioblastoma cells known to be positive for MGMT expression; (iii) LN229 are glioblastoma cells known to be negative for MGMT, and LN229-M are a subline infected with a construct expressing MGMT cDNA [44]. Original blots see File S1.

In view of the large differential in IC50s between NEO212 and TMZ when applied to some of the AML cell lines, we performed Western blot analysis of the levels of MGMT protein, the DNA repair protein known to affect the chemosensitivity of cells to TMZ. Figure 2 reveals that those 3 cell lines with high IC50s after TMZ treatment (HL60, KG1, THP1) also displayed pronounced expression levels of MGMT, whereas the two cell lines with low TMZ IC50s (U937 and 6D10) were negative for MGMT expression. While this close correlation of IC50s and MGMT levels was expected for TMZ, it did not emerge with NEO212. In the case of NEO212, MGMT-positive HL60 and KG1 cells were as effectively killed by NEO212 as were MGMT-negative U937 and 6D10 cells. In the case of MGMT-positive THP1 cells, treatment with NEO212 consistently exerted greater cytotoxic impact than TMZ, although these cells were somewhat less sensitive to NEO212 than the other four cell lines tested.

**Figure 3:**
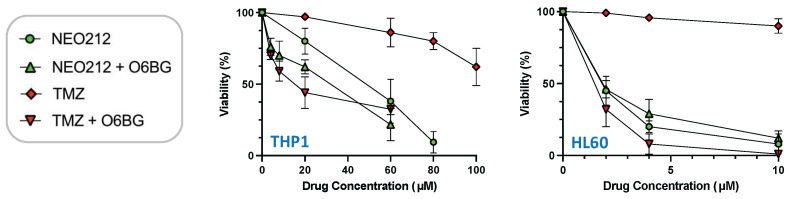
O6BG sensitizes cells to TMZ, but not to NEO212. THP1 and HL60 cells were exposed to increasing concentrations of NEO212 or TMZ in the presence or absence of 30 µM O6BG. After 5 days, cell viability was determined by MTT assay. All values were normalized to vehicle control.

**Figure 4:**
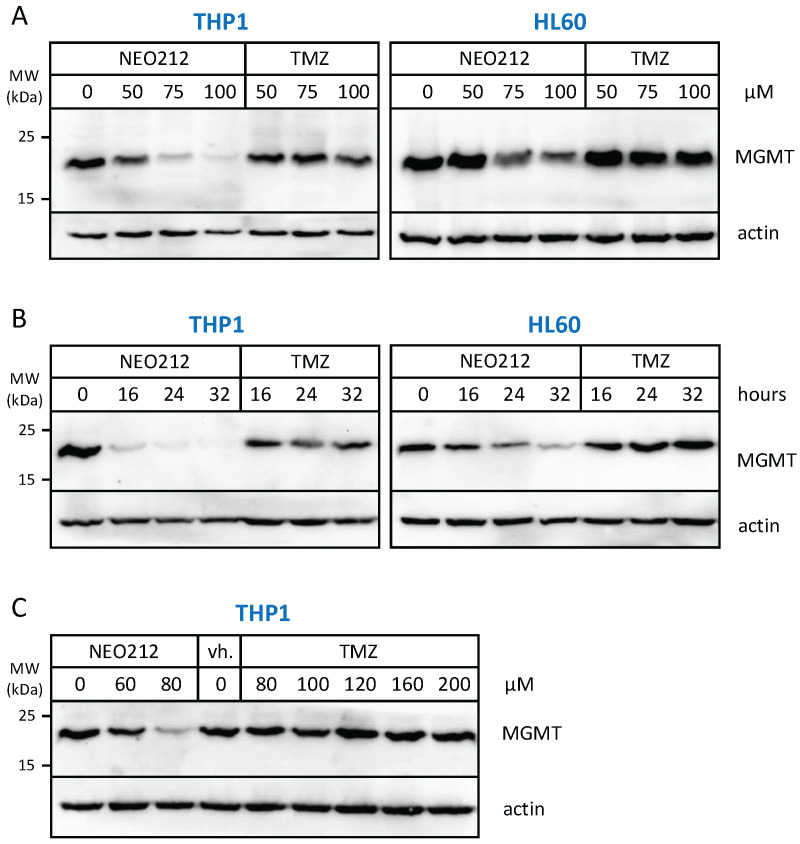
NEO212 treatment down-regulates MGMT protein levels. THP1 and HL60 cells were exposed to NEO212 or TMZ, and MGMT protein levels were determined by Western blot. (**A**) Cells treated with increasing drug concentrations were harvested after 24 h. (**B**) Cells treated with 80 µM of either drug were harvested at different time points thereafter. (**C**) Cells treated with increasing drug concentrations were harvested after 24 h. Original blots see File S1.

The relation to MGMT was investigated further by inclusion of O6-benzylguanine (O6BG), a potent small-molecule inhibitor of MGMT [45]. In the presence of O6BG, the cytotoxic potency of TMZ was hugely enhanced in MGMT-positive THP1 and HL60 cells, as would be expected (Figure 3). In comparison, the already higher cytotoxic potency of NEO212 was not substantially further enhanced in the presence of O6BG, indicating that MGMT was not able to confer chemoprotection against NEO212. To follow up on this result, we investigated MGMT protein levels in response to treatment with NEO212 and TMZ. As shown in Figure 4, treatment of cells with NEO212 resulted in striking down-regulation of MGMT protein levels, whereas treatment with TMZ had no effect. This down-regulation was somewhat more pronounced in THP1 cells as compared to HL60 and was detected as early as 16 h after the addition of 50 µM NEO212. In contrast, TMZ—even at greatly increased concentrations up to 200 µM—was unable to exert a detectable impact on MGMT protein levels (Figure 4C).

### 3.3. NEO212 Triggers Macrophage Differentiation

**Figure 5:**
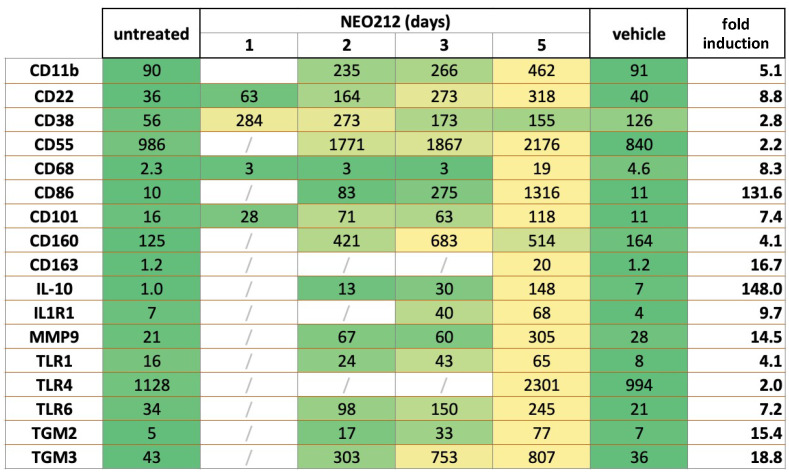
NEO212 stimulates expression of macrophage differentiation markers. AraC-resistant 6D10 cells were treated with 30 µM NEO212, harvested after 1, 2, 3, and 5 days, and processed for RNA-seq analysis. Comparator cell cultures were untreated cells or cells treated with vehicle DMSO for 5 days. Right column (fold induction) refers to the fold increase of the respective transcript after 5 days of drug treatment, as compared to untreated cells. Heatmap: green color shows pre-treatment levels and yellow color indicates an increase.

To gain some mechanistic insight into how NEO212 might achieve its potent anticancer effect against AML cells, we performed RNA-seq analysis of NEO212-treated 6D10 cells, which is an AraC-resistant subline of U937 cells. Cells were treated with NEO212 for 1, 2, 3, and 5 days and compared to cells that remained untreated or were treated with DMSO vehicle for 5 days. Among the many transcripts that were found to be affected by NEO212, there was a large group of significantly upregulated targets that were recognized as known markers of macrophage differentiation. Most of these transcripts slowly accumulated over the course of the 5-day analysis and showed their highest expression levels on day 5 after the addition of NEO212 (Figure 5). Fold induction levels varied from 2.0 to 4.1-fold for 5 of these transcripts, from 5.1 to 18.8-fold for 10 others, and from 131.6 to 142.0-fold for the remaining two. Besides providing evidence that NEO212 triggered differentiation of these AML cells along the macrophage pathway, these data also implied that treatment with NEO212 did not merely enact a rapid cytotoxic shut-down of cellular functions. Rather, even between days 3 and 5 after drug treatment, there was a substantial increase in the majority of these transcript levels, indicating continued cellular functioning.

**Figure 6:**
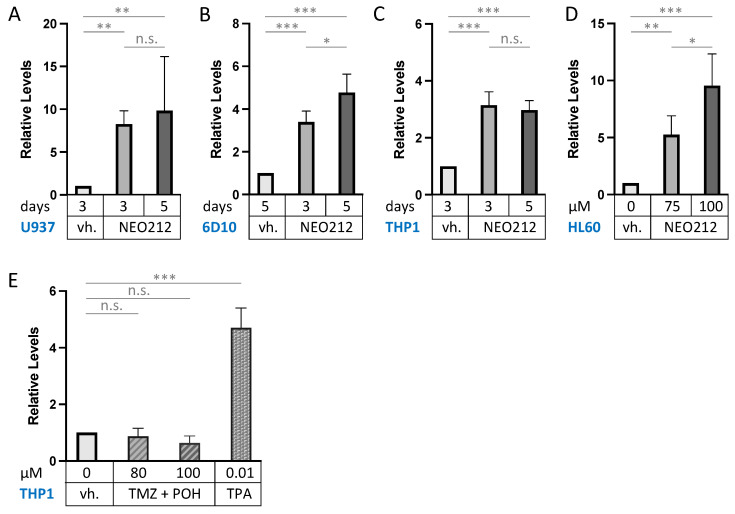
NEO212 increases CD11b mRNA levels. Drug-treated AML cell lines were harvested, and CD11b transcript levels were quantified by RT-qPCR. Control cells received vehicle (vh.) only (DMSO). (**A**) U937 cells were treated with 10 µM NEO212 for the indicated times. (**B**) 6D10 cells were treated with 10 µM NEO212. (**C**) THP1 cells were treated with 50 µM NEO212. (**D**) HL60 cells were treated with 10 µM NEO212. (**E**) THP1 cells were treated for 4 days with TMZ in combination with equimolar concentrations of POH. As a positive control, they were also treated with 10 nM TPA for 2 days. Asterisks: * = *p* < 0.05, ** = *p* < 0.01, *** = *p* < 0.001; n.s. = not significant.

CD11b (also called ITGAM: integrin subunit alpha M) is the most commonly used macrophage differentiation marker, and it was increased by 5.1-fold in the above transcriptomic analysis (Figure 5). We validated this effect in other AML cell lines. In addition to 6D10 cells, we extracted RNA from U937, HL60, and THP1 cells that had been treated with NEO212 for 3 or 5 days. The chosen NEO212 concentrations were based on the viability measurements in Figure 1 and were intended to be strongly growth-inhibitory and cytotoxic after 5 days; however, we also collected cells after 3 days, which represents an early time point with little drug toxicity at that time. RT-qPCR was performed on all samples, and it revealed significant (*p* < 0.01) induction of CD11b transcript in all cases, indicating a robust response that was consistently observed under different drug concentrations, different time points, and in different cell lines (Figure 6). As a positive control, we used the phorbol ester TPA, a well-established trigger of macrophage differentiation of AML cells [46], which in our hands increased CD11b expression as well (Figure 6E). However, treating cells with equimolar concentrations of TMZ mixed with POH was unable to trigger increased CD11b expression (Figure 6E), confirming that the combination of individual components that make up NEO212 is unable to mimic the effects of the conjugated product.

**Figure 7:**
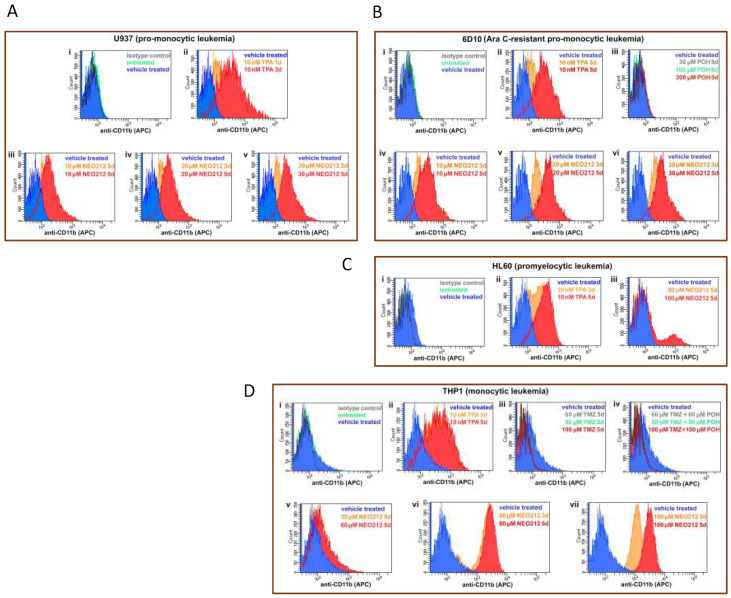
NEO212 stimulates cell surface presence of CD11b. AML cell lines were subjected to different treatments, followed by FACS analysis for CD11b positivity. The first square (top left, indicated with “i”) in each panel shows the following control histograms: untreated cells (green), vehicle treated cells (blue), and cells incubated with isotype control antibody (dark grey); as shown for each of the cell lines used, these negative controls showed no shift of the histograms. (**A**) U937 cells were treated with 10 nM TPA for 1 or 3 days (panel ii), or with 10, 20 and 30 µM NEO212 for 3 or 5 days (panels iii–v). (**B**) 6D10 cells were treated with TPA (ii), POH (iii), or with NEO212 (iv–vi). (**C**) HL60 cells were treated with TPA (ii) or with NEO212 (iii). (**D**) THP1 cells were treated with TPA (ii), TMZ (iii), TMZ combined with POH (iv), or with NEO212 (v–vii).

Increased CD11b expression could also be confirmed by detecting this transmembrane protein on the surface of intact cells by staining with anti-CD11b antibodies, followed by FACS analysis. As summarized in Figure 7, U937, AraC-resistant 6D10, HL60, and THP1 cells treated with NEO212 presented with a pronounced increase in their respective mean fluorescence intensity, indicating the increased presence of CD11b protein. This effect could be mimicked by using the positive control, TPA, but it was not achieved by treatment with monotherapy POH (Figure 7B), monotherapy TMZ, nor combination treatment with equimolar concentrations of TMZ + POH (Figure 7D).

### 3.4. NEO212 Exerts Striking Therapeutic Activity In Vivo

We next moved to in vivo investigations. We had shown previously that NEO212 exerted therapeutic activity against MGMT-negative AML cells after implantation into mice [38]. Based on the above in vitro results establishing that NEO212 exerted potent cytotoxic effects against MGMT-positive AML cells as well, we included such a model in our mouse studies. We used MGMT-positive HL60 cells and MGMT-negative, highly AraC-resistant 6D10 cells. Both cell lines were implanted into immuno-compromised mice, followed by treatment with NEO212 or vehicle as the negative control. NEO212 was given in cycles, where one cycle consisted of once-daily oral gavage for five consecutive days. This cycle was followed by a treatment holiday of several days up to a week, followed by one more (HL60 cells) or two more (6D10 cells) treatment cycles. Thereafter, there were no further treatments.

**Figure 8:**
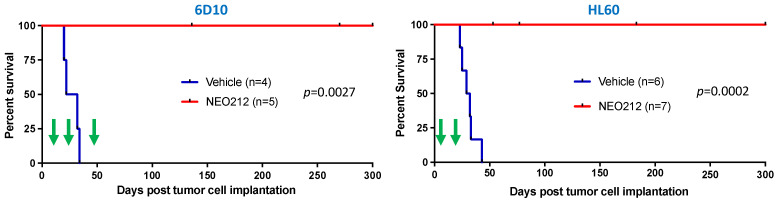
NEO212 exerts therapeutic activity in drug-resistant AML in vivo. AraC-resistant 6D10 cells (**left panel**) and TMZ-resistant HL60 cells (**right panel**) were implanted into immuno-deficient mice. Thereafter, animals were randomly separated into groups and subjected to vehicle or NEO212 treatment, as indicated by green arrows. The 6D10 model received three cycles of NEO212, starting on days 11, 25, and 48, respectively. The HL60 model received two cycles, starting on days 5 and 19, respectively. There were no further drug treatments beyond day 52 (6D10) or day 23 (HL60). In all cases, survival of animals was monitored and is presented as Kaplan–Meier plots. *p*-values shown represent statistical difference between NEO212-treated and vehicle-treated groups.

As presented in Figure 8, mice that received only vehicle treatment succumbed to disease rapidly and all of them died within 25–45 days after tumor cell implantation. In stark contrast, none of the NEO212-treated animals succumbed to disease for up to 300 days, at which time the experiment was terminated. This strikingly long survival, along with an apparent absence of any signs of disease, suggested that these animals might have been cured by NEO212 treatment. To gain further support for this view, we took blood from several of the mice in an effort to detect any remaining human tumor cells. We employed a PCR assay established by others [47], which measures the presence of human Alu sequences within the background of mouse tissue and has the sensitivity to detect one human cell among 100 million mouse cells. Using this assay, we were unable to detect human cells in mice that had been treated with NEO212. Thus, 300 days of survival in the absence of any signs of disease, combined with a lack of detectable human Alu sequences, indicated to us that the impact of NEO212 might indeed have been curative—and independent of the MGMT status of the tumor cells.

### 3.5. NEO212 Is Well Tolerated

**Figure 9:**
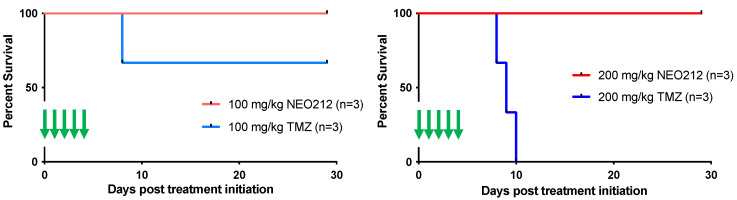
High-dose NEO212 does not impact survival. Three rats per group received one 5-day cycle of daily treatments with NEO212 or TMZ. Shown is survival of animals after treatment with 100 mg/kg per dose (**left panel**) and 200 mg/kg per dose (**right panel**). Green arrows indicate treatment days.

**Figure 10:**
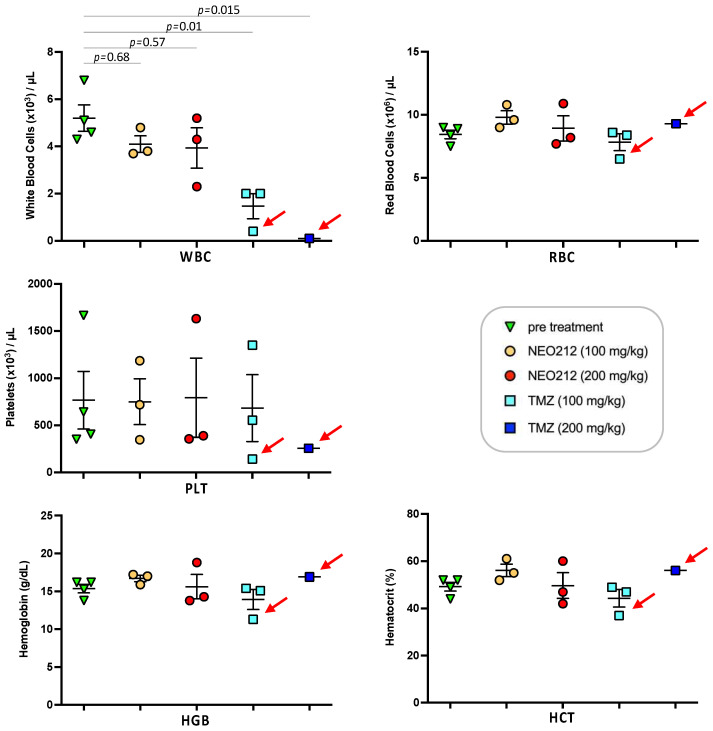
High-dose NEO212 shows no severe toxicity on blood parameters. Three rats per group received daily treatments with NEO212 or TMZ as described in the legend to Figure 9. Blood draw was performed seven days before the treatment cycle and four days after completion of the cycle. Shown is the number of white blood cells (WBC), red blood cells (RBC), and platelets (PLT), as well as the amount of hemoglobin (HGB) and the percentage of hematocrit (HCT). For the group of rats treated with 200 mg/kg TMZ, only one post-treatment value is available, because the other two animals in this group were found dead and blood draw was not possible (refer to Figure 9 for survival data). Red arrows point to animals that were moribund and did not recover from drug treatment. *p*-values: ordinary one-way ANOVA.

We next addressed the question of potential side effects of NEO212 treatment. In our earlier studies, we had established that NEO212 retains the alkylating potency of its TMZ subunit [38,48], and therefore myelosuppression might be of concern. However, our previous studies with NEO212 in mice were unable to detect significant alterations in white blood cell (WBC) counts or other markers of bone marrow toxicity; similarly, no changes in body weight or behavior, or any signs of pathological organ damage could be documented [6,7,38,44,49]. In an effort to approach the limits of NEO212 tolerability, we switched to rats, because this rodent model allowed us to administer substantially higher oral dosages of drug, far above what is needed for therapeutic purposes. As a meaningful standard, we applied TMZ in comparison. Three rats per group received one cycle (5-day repeat dosing) of 100 or 200 mg/kg NEO212 or TMZ per day. Blood was collected several days before the cycle and a few days afterwards. Throughout the treatment cycle and thereafter, all six rats treated with NEO212 continued to thrive without any clinical symptoms or changes in body weight or behavior. In contrast, all three rats treated with 200 mg/kg TMZ and one rat treated with 100 mg/kg TMZ died shortly after conclusion of the treatment cycle (Figure 9). Examination of blood from the moribund TMZ-treated rats showed signs of severe myelosuppression, as the number of white blood cells (WBC) was reduced by >90%. In comparison, no such dramatic reduction in WBC was seen in any of the NEO212-treated rats; while one of these animals showed a WBC count that was slightly below normal, WBC counts remained within the normal range in the other five rats (Figure 10). Furthermore, there were no changes in red blood cell counts, number of platelets, hemoglobin levels, and hematocrit values in these animals. Together, these results demonstrated that NEO212 was well tolerated even at 200 mg/kg, which represents a dose that is substantially higher than what was required to achieve an apparent AML cure in our mouse models.

## 4. Discussion

Our study employed preclinical AML models to investigate the anticancer effects of NEO212, a novel hybrid molecule generated by conjugating POH and TMZ. We established that NEO212 blocked in vitro proliferation of five different AML cell lines, including cells that are chemoresistant to AraC (6D10 cells) and three (HL60, KG1, THP1) that are chemoresistant to TMZ, based on their high expression levels of MGMT. Growth inhibition was accompanied by the emergence of numerous markers for macrophage differentiation. In two in vivo mouse models with chemoresistant cells (HL60 and 6D10), a few short cycles of oral NEO212 achieved long-term survival of all treated animals, where animals thrived for 300 days in the absence of detectable side effects of drug treatment and without any signs of remaining tumor cells. Toxicity studies in rats given NEO212 at doses that far exceeded therapeutically effective requirements revealed that this drug was exceedingly well tolerated over a wide therapeutic window.

MGMT repairs DNA by a “suicide” mechanism. It removes the methyl adduct resulting from alkylating agents at the O6-guanine position and transfers this moiety onto itself, thereby triggering degradation of the enzyme through the proteasome pathway. This process is stochiometric and therefore provides an indication of the severity of DNA methylation at the O6-guanine position. Our finding that NEO212 treatment results in diminished amounts of intracellular MGMT protein suggests that the drug quite potently methylates DNA at O6-guanine. Intriguingly, TMZ—which is well known as a methyl donor for O6-guanine—is unable to exert this down-regulatory effect on MGMT at comparable (or even double) concentrations (Figure 4). One explanation for this differential could be that more NEO212 than TMZ enters the cell, achieving greater numbers of methyl-O6-guanine moieties, which in return pose a greater workload for MGMT-mediated repair in NEO212-treated cells. Support for this model comes from our previous studies [48] that revealed the following. Once NEO212 is taken up by cells, it decays into its components TMZ and POH. Intriguingly, after treatment of cells with NEO212, intracellular TMZ concentrations are higher than after treatment of cells with TMZ, which is consistent with more extensive O6-guanine DNA methylation.

In the case of MGMT-negative cells, we found that TMZ was similarly effective as NEO212 (Figure 1), which seems to contradict the view that greater efficacy of NEO212 is due to its superior cell entry. While we are unable to offer a good explanation at this time, we speculate that this might be the result of an unusual cell type-specific effect. In the past, we have compared the cytotoxicity of NEO212 and TMZ in a large number of MGMT-negative cell lines from different tumor types, and in all cases we consistently found that the cytotoxic IC50 of NEO212 was significantly lower than the IC50 of TMZ [6,7,8,36,49]. The U937 cell line (and its AraC-resistant 6D10 subline) are a notable exception in this regard, which will require further investigation.

While its ability to alkylate O6-guanine suffices to explain the cytotoxic potency of NEO212, it is reasonable to assume that its capacity to induce macrophage differentiation, as discovered in this study, might contribute as well. It has been known for a long time that certain agents can stimulate AML cells to undergo macrophage differentiation, which is accompanied by proliferation arrest and eventual apoptosis [46,50,51]. For example, the phorbol ester TPA is a classic inducer of this process; it triggers proliferation arrest and induction of macrophage markers such as integrin CD11b [46,52,53]. After several days of sitting in this TPA-induced differentiated state, the cells slowly enter apoptosis and die. We have included TPA in several of our experiments as a positive control and observed many similarities to NEO212-treated cells, which include growth arrest, pronounced increase in a set of macrophage differentiation markers, and slow entry into apoptosis. Of note, past studies also showed that treatment with the organosulfur compound DMSO is able to trigger this differentiation process, and effective concentrations were reported in the range of 1.1 to 1.4% [54,55,56]. Because DMSO was used as a solvent for most of our drugs, it was critical to exclude that NEO212-induced differentiation was not caused by the DMSO vehicle in our experiments. While we did observe growth-inhibitory effects of DMSO starting at around 0.8% and becoming highly potent at 1.5%, we did not detect any such effects at 0.4% and below (Figure 1C). As DMSO concentrations in our experiments were kept below 0.2%, and because we used it as a comparator to untreated cells in all our experiments, we were able to exclude that the low DMSO concentrations used in our experiments had measurable biological activity.

Among the striking results of our study was the finding that NEO212 achieved an apparent cure of mice that harbored drug-resistant AML cells. None of the NEO212-treated animals succumbed to disease, but rather thrived until the pre-set endpoint of 300 days (Figure 8), which can be considered equivalent to 30 years in a human [57]. Our significant efforts at detecting any remaining human tumor cells in these mice were unsuccessful, underscoring our view that the activity of NEO212 was eradicative and curative. In comparison, we have previously applied NEO212 to solid tumor models that included glioblastoma, melanoma, breast cancer, lung cancer and others [6,7,8,36,49,58]. While NEO212 exerted significant therapeutic activity in all these in vivo models, we did not observe curative effects. AML therefore stands out as a cancer type where the application of NEO212 might unfold its greatest benefit for patients in the clinic. 

While the underlying mechanism for NEO212′s differential in vivo potency against different cancer types remains to be fully investigated, it is possible that it is based, at least in part, on the physiological differences between liquid and solid cancer types, primarily the effect of interstitial fluid pressure (IFP) present in solid tumors. It has been well recognized that the microenvironment of solid tumors commonly harbors elevated IFP, which is thought to be based on high cell density, abnormal extracellular matrix, vascular abnormalities, and poor venous and lymphatic drainage [59,60]. High IFP may impede therapeutic drug delivery to the tumor tissue, and it has been associated with lower treatment response and poor prognosis in several solid cancer types [59,60,61]. In comparison, liquid tumors such as AML—where tumor cells are distributed in the systemic circulation and where the vasculature of their bone marrow niche is different from the vasculature in other organs [62]—are more readily and more homogeneously accessible to drugs present in the blood stream. While this conjecture provides a reasonable rationale for NEO212′s high potency in the preclinical AML models used in our study, it still remains to be validated experimentally. Moreover, it is possible that AML cells are more susceptible to NEO212 because they harbor an inactive macrophage differentiation pathway that is not present in solid tumors; NEO212-inititated re-activation of this pathway, which is incompatible with further proliferation, might contribute an additional block to further tumorigenesis.

Noteworthy is the relatively early onset of treatment of mice with NEO212. In our in vivo models, vehicle-treated animals started to die beginning on Day 20, which necessitated to initiate the 5-day treatment cycles well before that. In the case of 6D10 AML cells, we started treatment on Day 11 (Figure 8) to ensure that we would at least be able to complete one full cycle of treatment before control animals would start to die. Within this context, a limitation of our study is that the extent of leukemogenesis was not characterized before the onset of drug (or vehicle) treatment. Although the systemic dissemination of tumor cells after i.v. or i.p. injections of AML cells into mice is well described in the literature [42,43], we did not perform our own analysis of the presence of AML in spleen, bone marrow and liver, which represent the typical target organs for AML spread. For this reason, the extent of leukemogenesis at the time of treatment is not entirely clear, and it is possible that NEO212 effects could have been predominantly preventive, rather than therapeutic. Nonetheless, leukemogenesis was confirmed in vehicle-treated mice at the time they became moribund (by observation of the typical hunched posture and detection of human Alu sequences in their blood, which indicated cause of death as leukemia). In comparison, none of the mice treated with NEO212 showed any behavioral signs of disease, and at the time of euthanasia on Day 300 no human cells could be detected in their blood.

A critical consideration for any cancer therapeutic approach is the concept of therapeutic window, i.e., the range of drug concentrations that can be given to achieve therapeutic benefit without causing unacceptable toxicity. In this regard, NEO212 appears to fare exceedingly well. In our AML mouse models, 25 mg/kg NEO212 achieved curative outcomes (Figure 8). At the same time, this dose did not cause any detectable side effects, as we have extensively documented in the past with the use of complete blood counts (CBC) with differential, blood chemistry and liver/kidney panels, histopathological organ analysis, or behavioral observations [6,7,38,44,49].

For future clinical trials, it would be helpful to know what type of potential side effects should be expected, and for alkylating agents the potential impact on the bone marrow is of particular interest. For this reason, we sought to increase NEO212 dosages with the intent to reach the maximally tolerated dose (MTD), which usually provides indicators of emerging toxicity. However, mice were not suitable to receive the increased volumes of drug that needed to be administered, and for that reason we switched to rats as an additional rodent model to explore the toxicity of NEO212. Yet, even at 200 mg/kg repeat dosing, the rats continued to thrive and no significant toxicities became apparent (Figure 9 and Figure 10). In all, these results point to an unusually large therapeutic window for NEO212, which is highly encouraging for its further development as an anticancer agent.

The study of side effects, in particular potential myelosuppression, represents a critical component of drug development, especially when it entails drugs with DNA alkylating potency [63,64]. Because NEO212 harbors this activity (Figure 4 and refs. [6,7,8]), we expended much effort to clarify its potentially suppressive impact on the bone marrow. As detailed above, however, the risk for this type of side effect appears quite low. Contrasting bone marrow suppression, another risk of alkylating agents is treatment-related AML (t-AML), a rare condition where AML arises secondary to treatment of other tumors with alkylating agents [65]. In the context of relapsed and refractory leukemia, however, this risk would be immaterial, as AML already has developed. In all, we conclude that NEO212 harbors significant therapeutic potential for AML in the absence of excessive side effects, and it should be considered for further development toward clinical testing. The fact that NEO212 has a large therapeutic index suggests that it might also be beneficial for combination therapy with current or new pharmacological anticancer agents, and this aspect will be the subject of future investigations.

## 5. Conclusions

Our study presents NEO212 as a novel agent with promise for the treatment of AML. Its mechanism of action involves forcing tumor cells into the macrophage differentiation pathway, which is incompatible with continued proliferation. In mouse models, an apparent cure of drug-resistant AML was achieved in the absence of detectable drug toxicity. In all, these results bode well for future clinical applications of NEO212, particularly in relapsed and refractory settings. Its oral mode of administration (i.e., in pill or capsule formulation) provides advantages over many other AML drugs that have to be delivered intravenously, which is extremely encouraging for the treatment of children, as 20% of AML patients are in the pediatric population. We conclude that further development of NEO212 toward clinical use is warranted.

## Data Availability

All data is contained within this article.

## Supplementary Materials

The following supporting information can be downloaded at: https://www.mdpi.com/article/10.3390/cancers14246065/s1, File S1: Original blots of Figure 2 and Figure 4.
