# Supplementary figures and images for: NEO212, a Perillyl Alcohol-Temozolomide Conjugate, Triggers Macrophage Differentiation of Acute Myeloid Leukemia Cells and Blocks Their Tumorigenicity
Source: Cancers (Basel). 2022 Dec 9;14(24):6065. doi: 10.3390/cancers14246065 (PMC9776529; doi:10.3390/cancers14246065)

## Supplementary Materials

(original images of Western blots)

Figure 2

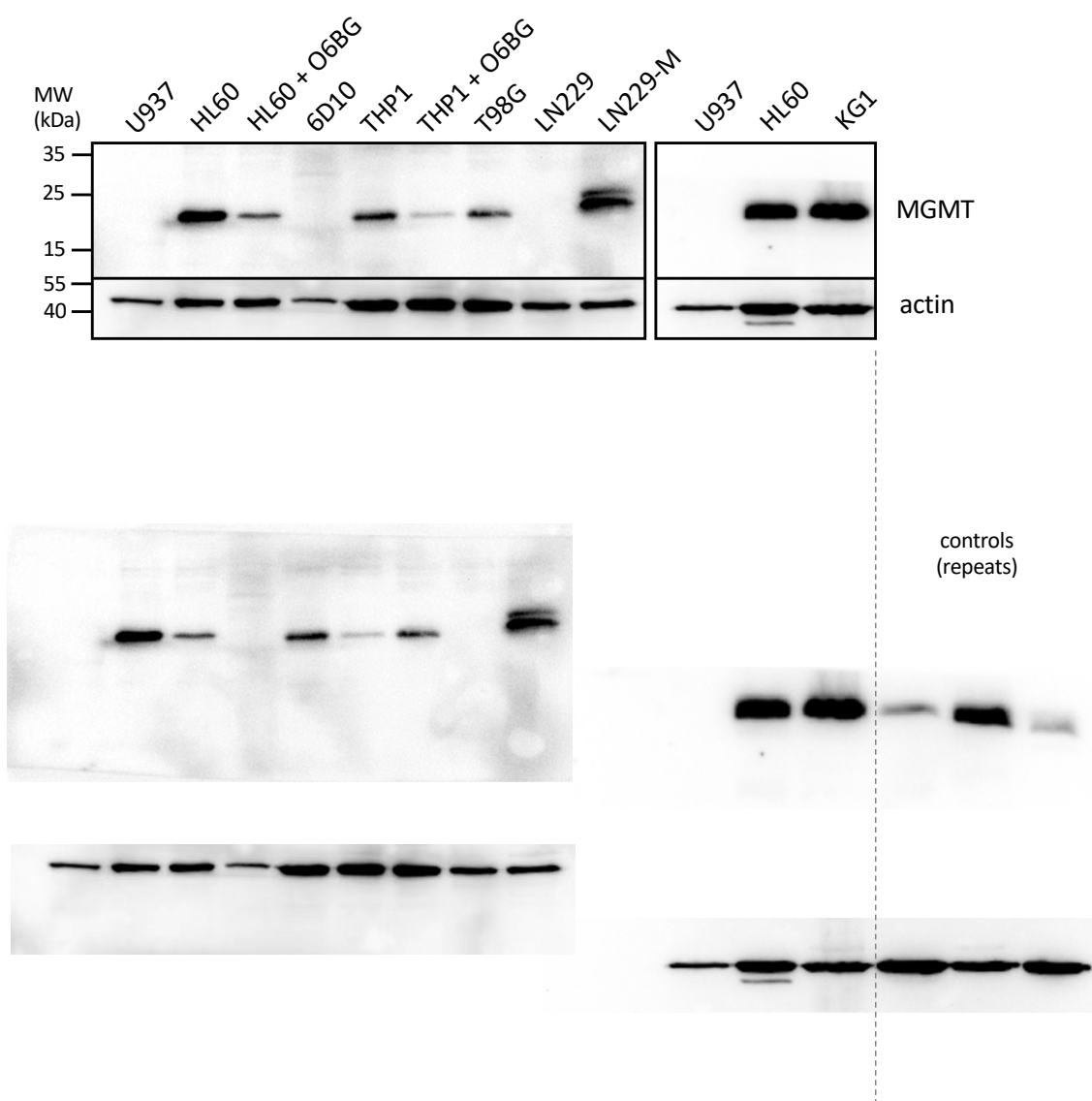

Figure 4 A

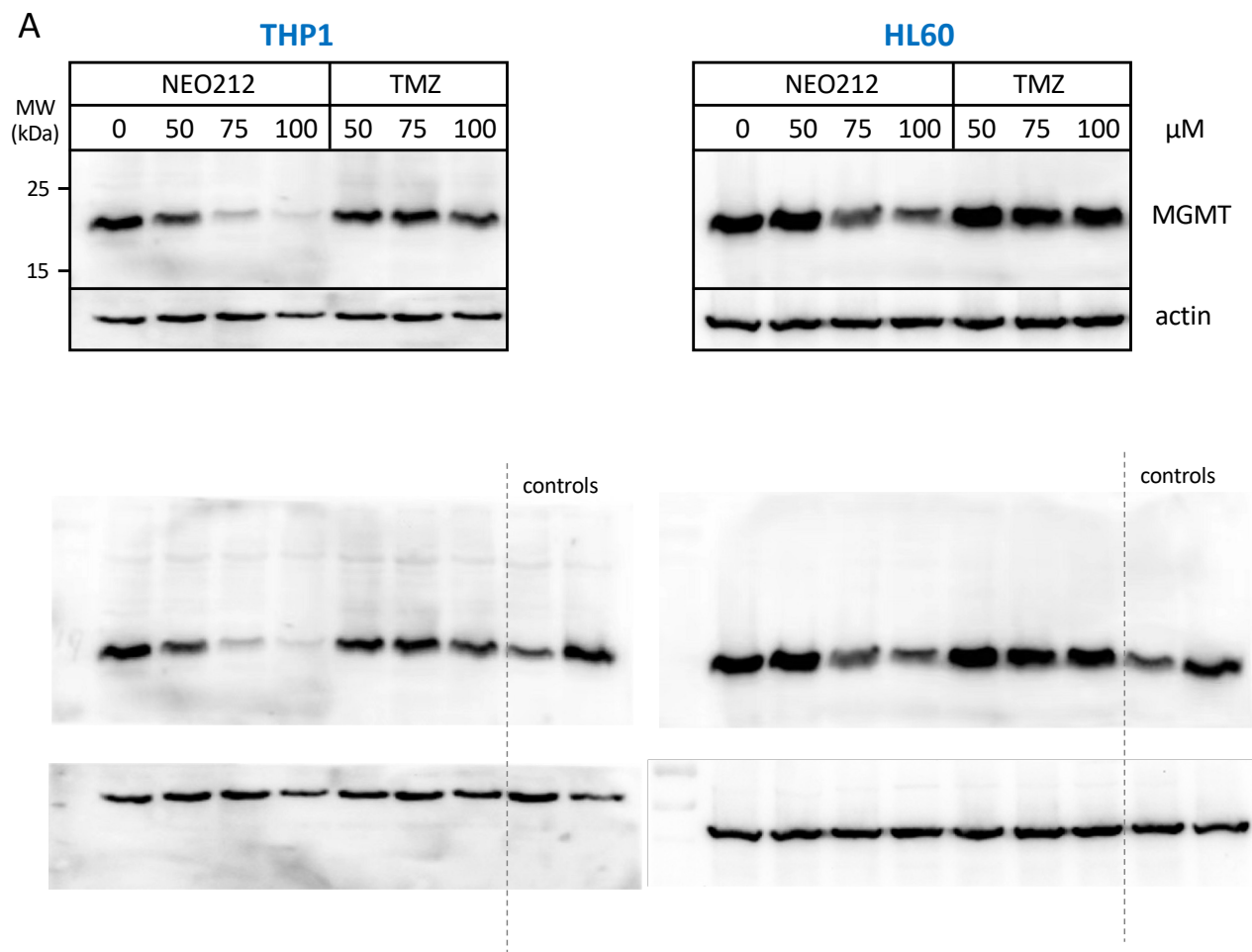

Figure 4 B

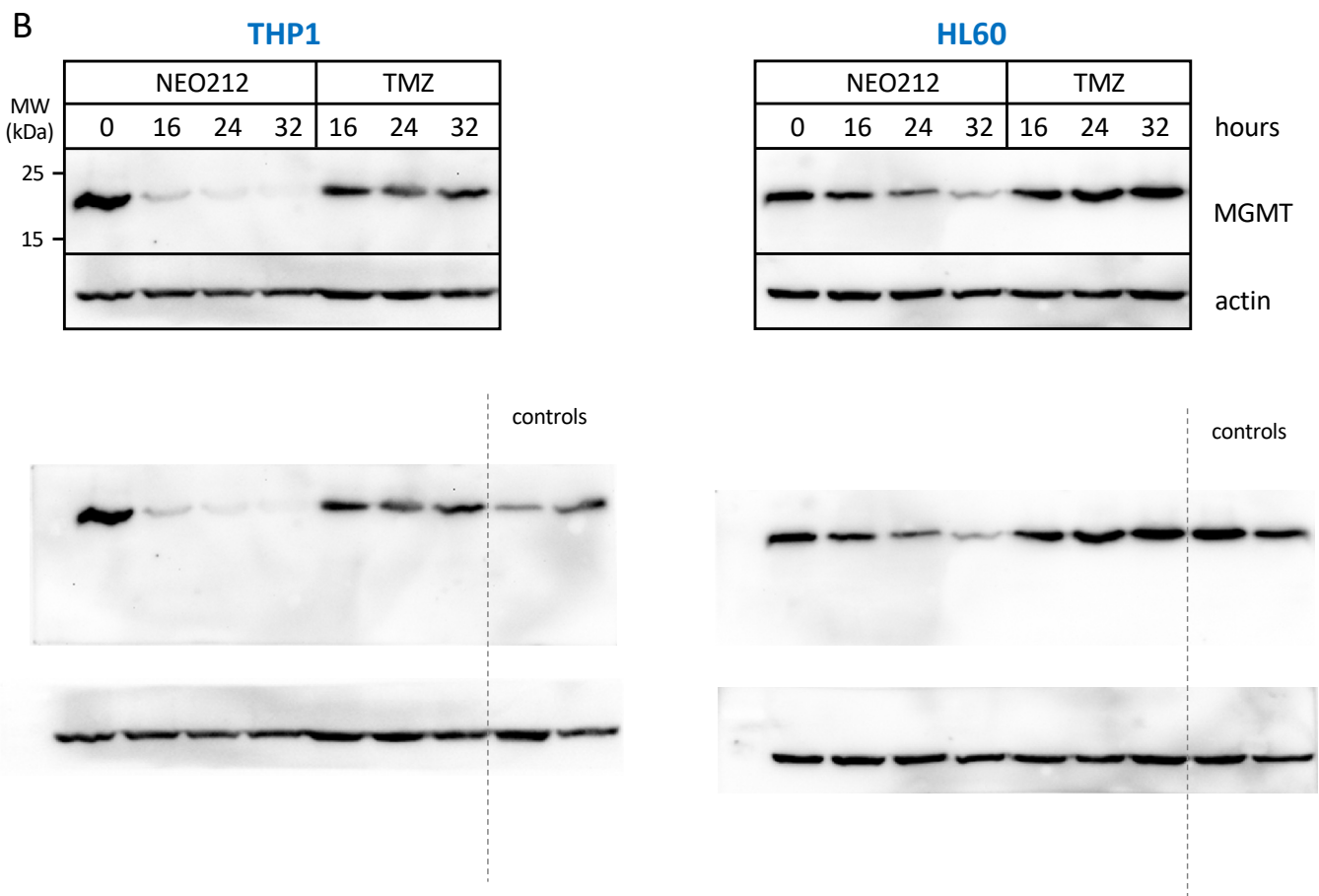

Figure 4 C

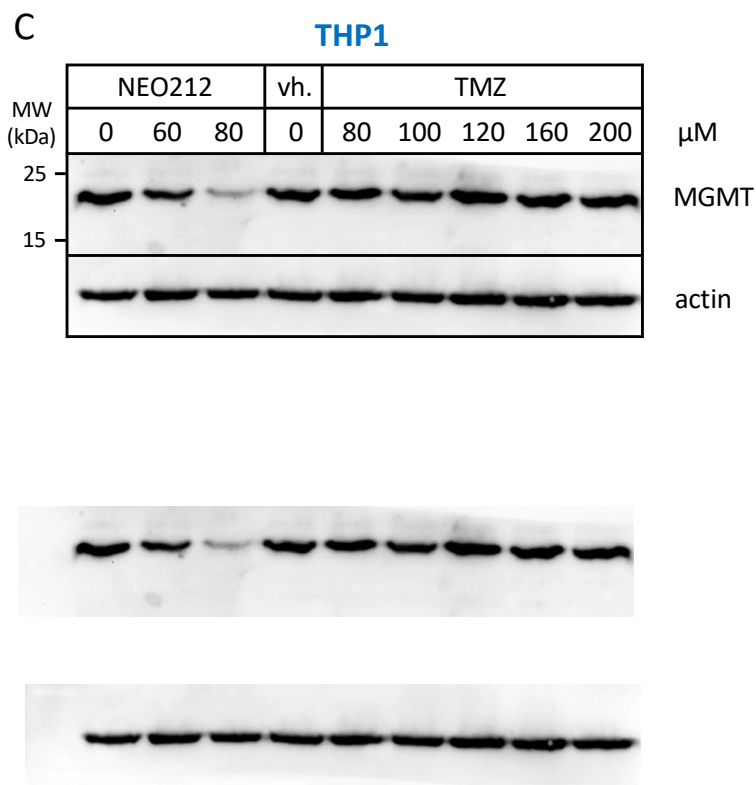

Supplement: Supplementary file 1 [file cancers-14-06065-s001.zip › cancers-2002579-supplementary.pdf]
